# Transcriptome analysis based detection of *Plasmodium falciparum* development in *Anopheles stephensi* mosquitoes

**DOI:** 10.1038/s41598-018-29969-4

**Published:** 2018-08-01

**Authors:** Miranda S. Oakley, Nitin Verma, Timothy G. Myers, Hong Zheng, Emily Locke, Merribeth J. Morin, Abhai K. Tripathi, Godfree Mlambo, Sanjai Kumar

**Affiliations:** 1 Laboratory of Mucosal Pathogens and Cellular Immunology, Division of Bacterial, Parasitic, and Allergenic Products, Office of Vaccines Research and Review, Food and Drug Administration, Silver Spring, MD USA; 2 Laboratory of Emerging Pathogens, Division of Emerging and Transfusion Transmitted Diseases, Office of Blood Research and Review, Center for Biologics Evaluation and Research, Food and Drug Administration, Silver Spring, MD USA; 3 Microarray Research Facility, Research and Technologies Branch, National Institutes of Health, Bethesda, MD USA; 4 PATH’s Malaria Vaccine Initiative, Washington, DC USA; 5 Johns Hopkins Bloomberg School of Public Health, Department of Molecular Microbiology and Immunology, Baltimore, MD USA

## Abstract

The *Plasmodium* life cycle within the mosquito involves the gamete, zygote, motile ookinete, and the oocyst stage that supports sporogony and sporozoite formation. We mapped the *P. falciparum* transcriptome as the parasite progresses through the oocyst stage of development on days 2, 4, 6, and 8 post-*P. falciparum* infectious blood meal. Through these genomic studies, we identified 212 novel transmission stage biomarkers including genes that are developmentally expressed at a single time point and genes that are pan-developmentally expressed at all four time points in *P. falciparum* oocysts. Validation of a small subset of genes at the transcriptional and translational level resulted in identification of a signature of genes/proteins that can detect parasites within the mosquito as early as day 2 post-infectious blood meal and can be used to distinguish early versus late stage *P. falciparum* oocyst development in the mosquito. Currently, circumsporozoite protein (CSP), which is detectable only after day 7 post-infection, is the only marker used for detection of *P. falciparum* infection in mosquitoes. Our results open the prospect to develop a non-CSP based detection assay for assessment of *P. falciparum* infection in mosquitoes and evaluate the effect of intervention measures on malaria transmission in an endemic setting.

## Introduction

Malaria remains one of the most serious global public health challenges. Nonetheless, between 2000 and 2014, malaria incidence rates have decreased by 37% and mortality rates by 60%^1^. However, more recent years have seen a decline in the success of malaria control efforts. In the year 2015, 211 million malaria cases and in 2016, 216 million cases of malaria were reported (WHO Report 2017). Several adversarial factors such as population growth and climate change and a need for sustained intervention efforts by international aid groups and government agencies in endemic countries may be accountable for the stalling of progress in the fight against malaria. These results also indicate that more novel approaches and tools to assess the effect of intervention measures may be needed to cause a further reduction in malaria infection rates in areas of transmission.

The *Plasmodium* life cycle relies on a mammalian host for asexual replication to generate intra-erythrocytic forms and a mosquito vector for further transmission which includes sexual reproduction and exchange of genetic materials. The sexual stage in the mosquito commences when an *Anopheles* mosquito bites a gametocyte-carrying infected host and then the parasite undergoes the following developmental phases: gametogenesis (differentiation of male and female gametocytes into male and female gametes); fertilization leading to formation of a diploid zygote; motile ookinete; and the oocyst^2^.

The duration of the mosquito cycle varies between *Plasmodium* species; for *P. falciparum* it lasts for approximately 21 days. The oocyst stage begins at day 2 post-fertilization, when the ookinete traverses the peritrophic membrane and develops into oocysts in the basal lamina of the midgut^3^. The oocyst phase lasts for approximately 10–12 days and is characterized by extensive sporogony resulting in the production of several hundreds to thousands of sporozoites which eventually leave the midgut and travel to the salivary gland of the mosquito and attain their infectious form^4,5^.

During the past decades, there has been a significant improvement in our knowledge of the molecular processes and parasite molecules involved in gametogenesis, fertilization, and ookinete formation in mosquitoes^6–8^. However, the molecular events and parasite molecules that govern developmental progression of the midgut oocysts are poorly understood. Circumsporozoite protein (CSP), a sporozoite surface protein that is currently the only marker available for use in mosquito-stage immunological assays^9^, can first be detected in oocysts on day 7 post-blood meal^10^. The earliest microscopic detection of developing oocysts is only feasible after day 6 post blood-meal^3^. Thus, there is an unmet need for the discovery of novel parasite markers that can be used for *Plasmodium* detection during the early stages of parasite development in infected mosquitoes. Earlier detection of a *Plasmodium* infection during the developmental cycle in the *Anopheles* mosquito midgut would provide a more accurate picture of the mosquito infectivity rate and help measure the effectiveness of intervention tools such as seasonal malaria chemoprevention programs, vaccines, bed-nets, and insecticides in endemic areas.

In this study, we characterized the transcriptional profile of *P. falciparum* developmental progression through the *Anopheles stephensi* midgut on days 2, 4, 6 and 8 post-blood meal, identified a genetic signature that detects presence of the parasite in the mosquito, and developed an immunological assay based on the detection of individual or a combination of four antigens. To our knowledge, this is the first study to demonstrate detection of *P. falciparum* in mosquitoes as early as day 2 through day 8 post-blood meal. These results support development of multiplex genetic and immunological assays that can detect *Plasmodium* at all developmental stages within infected mosquitoes for surveillance and drug and vaccine efficacy studies.

## Results

### Identification of parasite biomarkers of malaria transmission by microarray analysis of the *P. falciparum* transcriptome as the parasite developmentally progresses through the mosquito

Microarray analysis was performed in four mechanical replicates using RNA purified from uninfected mosquito midguts versus infected mosquito midguts (2, 4, 6, and 8 days post-infection). Twenty midguts were pooled per time point and the average oocyst burden of mosquito midguts at day 8 was 64 oocysts/midgut. Importantly, equivalent molar amounts of cy3 (uninfected mosquito midguts) and cy5 (infected mosquito midguts) labeled cRNA was hybridized to a *P. falciparum* microarray chip (Agilent Technologies, Santa Clara, CA).

We used five-fold differential expression and a p value less than 0.05 as criteria for significance. For day 2 post-infection, the input data was 4 arrays and 5777 genes. 5771 genes were eliminated for having a fold change ratio less than five-fold resulting in a dataset containing 6 differentially expressed genes. For day 4 post-infection, the input data was four arrays and 5762 genes. 5702 genes were eliminated for having a fold change ratio less than five-fold and one gene was eliminated for having a p value ≥ 0.05 resulting in a dataset containing 59 differentially expressed genes. For day 6 post-infection, the input data was four arrays and 5732 genes. 5612 genes were eliminated for having a fold change ratio less than five-fold resulting in a dataset containing 120 differentially expressed genes. For day 8 post-infection, the input data was four arrays and 5766 genes. 5614 genes were eliminated for having a fold change ratio less than five-fold and one gene was eliminated for having a p value ≥ 0.05 resulting in a dataset containing 152 differentially expressed genes. In total, we have identified 212 novel parasite biomarkers of transmission stage malaria.

### Functional annotation of putative parasite biomarkers of transmission stage malaria

Bioinformatic analysis was applied to the dataset to systematically classify the 212 identified parasite biomarkers of transmission stage malaria into functional categories (Table 1 and Supplemental Table 1). Among the 212 differentially expressed genes, 28 (13.2%) of the genes encode conserved proteins that have no known function. Remarkably, 6 genes (*Pf*. von Willebrand factor A domain-related protein*, Pf*. histone H2A*, Pf*. circumsporozoite and TRAP-related protein, *Pf*. histone H3 variant*, Pf*. dihydrofolate synthase/folylpolyglutamate synthase, and *Pf*. DNA/RNA-binding protein Alba 1) had expression changes as early as day 2 post-infection. In addition, although 128 (60.4%) of the significant genes were unique to a particular developmental stage, a large subset of these genes had abundance changes at more than one time point. Importantly, 3 genes (*Pf*. DNA/RNA-binding protein Alba 1, *Pf*. Histone H2A, and *Pf*. Histone H3 variant) had significant expression at all four measured time points (days 2, 4, 6, and 8) and 34 genes had significant expression at three time points (days 4, 6, and 8) (Table 1).

**Table 1:** Biological functions of a subset of transmission stage specific parasite genes in our dataset.

Protein description	Gene	Fold Change
**Chaperone/Protein stability**		
Endoplasmin	PF3D7_1222300	Day 6 and 8
Heat shock 70	PF3D7_0818900	Day 4, 6, and 8
Heat shock protein 70	PF3D7_0917900	Day 4, 6, and 8
Heat shock protein 90	PF3D7_0708400	Day 4, 6, and 8
Heat shock protein DNAJ homologue Pfj4	PF3D7_1211400	Day 8
HSP40, subfamily A	PF3D7_1437900	Day 6 and 8
Hsp70/Hsp90 organizing protein	PF3D7_1434300	Day 6
co-chaperone p23	PF3D7_1453700	Day 4, 6, and 8
T-complex protein 1 subunit ε	PF3D7_0320300	Day 6 and 8
T-complex protein 1 subunit β	PF3D7_0306800	Day 6 and 8
T-complex protein 1 subunit α	PF3D7_1132200	Day 8
T-complex protein 1 subunit δ	PF3D7_1357800	Day 6
Tubulin-specific chaperone a	PFA_0460c	Day 8
**Cytoskeleton**		
Actin I	PF3D7_1246200	Day 4, 6, and 8
Actin-depolymerizing factor 1	PF3D7_0503400	Day 8
Tubulin β chain	PF3D7_1008700	Day 8
Nuclear polyadenylated RNA-binding protein NAB2	PF3D7_0623100	Day 8
**Cell Cycle**		
Cell division cycle protein 48 homologue	PF3D7_0619400	Day 8
Proliferating cell nuclear antigen 1	PF3D7_1361900	Day 4 and 8
**Chromatin**		
CCR4-NOT transcription complex subunit 5	PF3D7_1006100	Day 6
**DNA metabolism**		
ATP-dependent RNA helicase DDX5	PF3D7_1445900	Day 6
RuvB-like helicase 2	PF3D7_1106000	Day 6
**RNA metabolism**		
Splicing factor 3B subunit 6	PF3D7_1224900	Day 6
Splicing factor 1	PF3D7_1321700	Day 8
serine/arginine-rich splicing factor 1	PF3D7_0517300	Day 6
Histone H2A	PF3D7_0617800	Day 2, 4, 6, and 8
Histone H2A variant	PF3D7_0320900	Day 4 and 8
histone H2B variant	PF3D7_0714000	Day 8
histone H2B	PF3D7_1105100	Day 4, 6, and 8
Histone H3	PF3D7_0610400	Day 8
Histone H3variant	PF3D7_0617900	Day 2, 4, 6, and 8
Histone H4	PF3D7_1105000	Day 4, 6, and 8
Pseudouridine synthase	PF3D7_0219500	Day 6
ATP-dependent RNA helicase DDX23	PF3D7_0518500	Day 8
**Ubiquitin and proteasome**		
26S protease regulatory subunit 4	PF3D7_1008400	Day 8
26S protease regulatory subunit 10B	PF3D7_1306400	Day 4, 6, and 8
26S proteasome regulatory subunit RPN8	PF3D7_0912900	Day 4 and 6
Beta3 proteasome subunit	PFA_0400c	Day 6
Proteasome subunit β type-4	PF3D7_0803800	Day 8
26S proteasome regulatory subunit RPN10	PF3D7_0807800	Day 8
**Transcription**		
DNA-directed RNA polymerase 2	PFA_0505c	Day 8
**Translation**		
60S ribosomal protein L36	PF3D7_1109900	Day 6 and 8
Elongation factor 1 (EF-1)	PF3D7_0319600	Day 6
Elongation factor 1-gamma	PF3D7_1338300	Day 4, 6, and 8
Elongation factor 2	PF3D7_1451100	Day 8
Elongation factor-1 alpha	PF3D7_1357100	Day 4 and 8
Eukaryotic translation initiation factor 5a	PF3D7_1204300	Day 4, 6 and 8
Eukaryotic translation initiation factor 2 subunit alpha	PF3D7_0728000	Day 8
Eukaryotic translation initiation factor 3 subunit B	PF3D7_0517700	Day 6
Eukaryotic translation initiation factor 4 gamma	PF3D7_1312900	Day 6
Peptidyl-prolyl cis-trans isomerase	PF3D7_0322000	Day 4, 6, and 8
Protein disulfide isomerase	PF3D7_0827900	Day 6 and 8
60S ribosomal protein L29	PF3D7_1460300	Day 6
Translation initiation factor IF-2	PF3D7_0607000	Day 8
Translation initiation factor SUI1	PF3D7_1243600	Day 4 and 8
40S ribosomal proteins	19	
60S ribosomal proteins	31	
**Protein processing**		
Peptide chain release factor subunit 1	PF3D7_0212300	Day 6
Signal peptide peptidase	PF3D7_1457000	Day 8
**Signal transduction**		
Casein kinase 2, alpha subunit	PF3D7_1108400	Day 8
GTPase-activating protein	PF3D7_0904000	Day 4, 6, and 8
Phosphoenolpyruvate carboxykinase	PF3D7_1342800	Day 8
Ras-related protein Rab-11a	PF3D7_1320600	Day 6
Ran-specific GTPase-activating protein 1	PFD0950w	Day 4 and 6
Serine/threonine protein phosphatase PP1	PF3D7_1414400	Day 8
**Metabolism**		
1-cys peroxiredoxin	PF3D7_0802200	Day 6
3-oxoacyl-(acyl-carrier protein) reductase	PF3D7_0922900	Day 8
protein DJ-1	PF3D7_0627500	Day 8
Adenosine deaminase	PF3D7_1029600	Day 8
Adenosylhomocysteinase	PF3D7_0520900	Day 8
ATP synthase F1, alpha subunit	PF3D7_0217100	Day 6
Cytochrome c oxidase subunit 2	PF3D7_1430900	Day 8
Dihydrofolate synthase/folylpolyglutamate synthase	PF3D7_1324800	Day 2
Fumarate hydratase	PF3D7_0927300	Day 6
NADP-specific glutamate dehydrogenase	PF3D7_1430700	Day 8
Glyceraldehyde-3-phosphate dehydrogenase	PF3D7_1462800	Day 6 and 8
Hypoxanthine phosphoribosyltransferase	PF10_0121	Day 8
L-lactate dehydrogenase	PF3D7_1324900	Day 4, 6, and 8
Malate dehydrogenase	PF3D7_0618500	Day 8
Phosphoethanolamine N-methyltransferase	PF3D7_1343000	Day 8
Phosphoglycerate mutase	PF3D7_1120100	Day 6
Purine nucleoside phosphorylase	PF3D7_0513300	Day 4, 6, and 8
Ribonucleoside-diphosphate reductase, large subunit	PF3D7_1437200	Day 8
S-adenosylmethionine decarboxylase-ornithine decarboxylase	PF3D7_1033100	Day 4 and 6
S-adenosylmethionine synthetase	PF3D7_0922200	Day 4, 6, and 8
Transketolase	PF3D7_0610800	Day 6
Cytochrome b-c1 complex subunit 7	PF3D7_1012300	Day 6 and 8
**Trafficking**		
Vesicle-associated membrane protein	PF3D7_1439800	Day 8
Nuclear transport factor 2	PF3D7_1412300	Day 8
AP-1 complex subunit mu-1	PF3D7_1311400	Day 8
**Known Transporters**		
ADP/ATP transporter on adenylate translocase	PF3D7_1037300	Day 6 and 8
Major facilitator superfamily-related transporter	PF3D7_0914700	Day 4, 6, and 8
**Surface/exported proteins**		
Asparagine-rich antigen	PF3D7_1110400	Day 6 and 8
Asparagine-rich antigen Pfa35-2	PFA_0280w	Day 8
Circumsporozoite (CS) protein	PF3D7_0304600	Day 8
Exported protein 1	PF3D7_1121600	Day 6 and 8
CSP and TRAP-related protein	PF3D7_0315200	Day 2
PHF5-like protein	PF3D7_1018500	Day 6
Antigen UB05	PF3D7_1038000	Day 4
Von Willebrand factor A domain-related protein (WARP)	PF3D7_0801300	Day 2
Erythrocyte membrane-associated antigen	PF3D7_0703500	Day 6 and 8
**Miscellaneous (selected)**		
Conserved *Plasmodium* protein	PF3D7_0931300	Day 6 and 8
superoxide dismutase [Fe]	PF3D7_0814900	Day 6
Glideosome-associated protein 45	PF3D7_1222700	Day 8
UVB-resistance protein UVR8 homologue	PF3D7_1137400	Day 8
Plasmodium repeat_MYXSPDY protein	PF10_0383	Day 8

### Correlation of expression measured by microarray versus quantitative real time PCR (qPCR)

**Figure 1:**
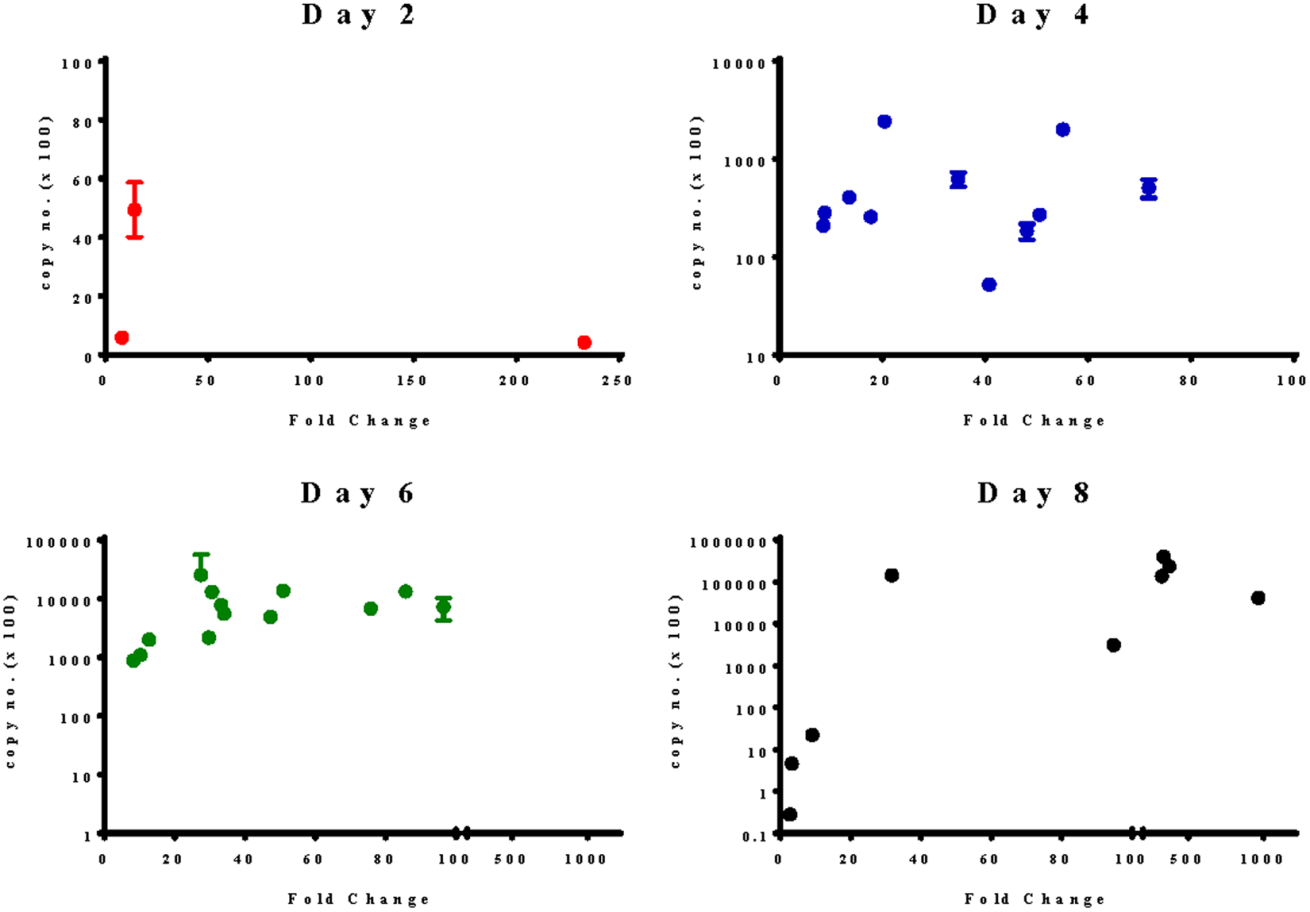
Correlation of transcriptional expression measured by microarray versus qPCR. 17% (36 genes) of the 212 novel parasite biomarkers of transmission stage malaria identified by microarray was subjected to validation by qPCR using primers specific for the gene of interest and RNA template isolated from infected mosquitoes. Each dot represents an individual gene in the microarray dataset. Correlation between fold change by microarray (x axis) versus copy number ± standard error by qPCR (y axis) increases as the parasite developmentally progresses through the mosquito. Correlation was calculated using the Spearman’s ρ nonparametric test. Error bars indicate standard deviation for qPCR.

RNA purified from uninfected mosquito midguts (cy3) and infected mosquito midguts (cy5) was co-hybridized to a microarray chip specific for the *P. falciparum* genome. A limitation of this experimental approach is that the absence of *P. falciparum* RNA in the cy3 channel may result in an increased rate of false positives. To ascertain whether the transcription level determined by microarray was a true measure of gene expression rather than an artifact introduced by the experimental design, a large proportion (17%) of the dataset of 212 genes was subjected to validation by qPCR using primers specific for the gene of interest. Correlation of fold change by microarray versus copy number by qPCR was then assessed (Fig. 1). Interestingly, the correlation between results obtained by microarray versus qPCR increased for later stages of the parasite development in the mosquito. There was no significant correlation (p = 0.58) between fold change determined by microarray versus copy number obtained by qPCR for genes significantly expressed by microarray on day 4 post-infection that were subsequently subjected to qPCR (n = 11). The correlation approached statistical significance (p < 0.06) for the subset of genes (n = 13) that were assessed on day 6 post-infection and there was a highly significant correlation (p < 0.02) for the subset of genes (n = 10) evaluated on day 8 post-infection. An increase in the accuracy of the microarray assay as the parasite developmentally progresses through the mosquito may be a consequence of an increase in the parasite: mosquito ratio of template RNA extracted from infected mosquito midguts.

### Validation of novel biomarkers of transmission stage malaria by qPCR

We selected a subset of candidate detection markers and compared their expression to *Pf*.CSP by qPCR during *P. falciparum* developmental progression (day 0 (uninfected), 6 hours, day 1, day 2, day 4, day 6, day 8) in *A. stephensi* mosquitoes in two independent experiments using different sets of mosquito midgut samples. The results of this qPCR experiment can be found in Table 2. The criteria for selection was based on the degree and duration of parasite gene expression in the mosquito determined by microarray (Table 1) and the correlation between fold change by microarray versus copy number by qPCR (Fig. 1). Primers used to perform this qPCR experiment can be found in Supplemental Table S2. *Pf*.CSP was detected as early as 6 hours post-infection (51.5 copies) and there was a dose dependent increase in expression that peaked on day 8 post-infection (1,620,000 copies). Remarkably, three genes (*Pf*.H2B, *Pf*.GAPDH, and *Pf*.TRP1) had expression that was higher than *Pf*.CSP throughout the course of the transmission stage of the parasite with *Pf*.H2B demonstrating the highest level of expression; *Pf*.H2B (4,620,000) had 2.85 fold more mRNA transcripts than *Pf*.CSP (1,620,000) on day 8 post-infection. Furthermore, although *Pf*.p23 and *Pf*.RACK had expression that was slightly lower than *Pf*.CSP on day 8 post-infection, the expression of both genes was significantly higher on days 1, 2, 4, and 6 post-infection compared to *Pf*.CSP. On day 6 post-infection, *Pf*.p23 (500,000) and *Pf*.RACK (386,000) had 7.5 and 5.8 fold more mRNA transcripts than *Pf*.CSP (66,800), respectively (Table 2).

**Table 2:** Measurement of expression of a subset of biomarkers by qPCR over time during the transmission stage of the parasite within the mosquito.

Gene	PlasmoDB ID	Day 0	6 hours	Day 1	Day 2	Day 4	Day 6	Day 8
Conserved	PF3D7_0931300	5	8.9	36	NA	240	18,500	1,690,000
CSP	PF3D7_0304600	0	51.5	65.8	416	1740	66,800	1,620,000
DHFS-FPGS	PF3D7_1324800	37.9	58.7	55	13,700	6970	62,800	192,000
ALBA1	PF3D7_0814200	0	4	0	341	1660	13,700	44,400
GAPDH	PF3D7_1462800	0	111	716	6020	84,800	1,030,000	3,250,000
H2B	PF3D7_1105100	0	2430	26,200	105,000	276,000	1,420,000	4,620,000
P23	PF3D7_1453700	0	70	592	10,100	61,500	500,000	1,080,000
RACK	PF3D7_0826700	0	35	314	4840	66,500	386,000	989,000
TRP1	PF3D7_1438900	0	111	915	5250	48,100	445,000	1,710,000
WARP	PF3D7_0801300	0	312	300	204,000	14,000	6390	11,900

Values shown represent mRNA copy number per 100 ng template RNA.

### Characterization of *P. falciparum* recombinant proteins and their respective monoclonal antibodies

In order to measure expression at the protein level of candidate parasite detection markers within the mosquito, we produced recombinant proteins that encode the *Pf*. conserved *Plasmodium* protein (PF3D7_0931300), *Pf*.RACK (PF3D7_0826700), *Pf*.TRP1 (PF3D7_1438900) and *Pf*.WARP (PF3D7_0801300) genes and respective monoclonal antibodies against these recombinant proteins. The *Pf*. conserved *Plasmodium* protein, *Pf*.RACK, *Pf*.TRP1 and *Pf*.WARP recombinant proteins consist of 305, 322, 194 and 265 amino acid residues with an additional sequence to include the hexa-histidine tag and a spacer which gives calculated molecular weights of 37, 38, 24 and 32 kDa, respectively. Protein characterization was performed by 4–12% Sodium Dodecyl Sulfate-Poly Acrylamide Gel Electrophoresis (SDS-PAGE) following Coomassie blue staining (SimplyBlue SafeStain; Thermo Fisher Scientific, Waltham, MA) (Supplemental Figure S1). Results showed that the purified proteins were highly pure with no visible contaminating bands. Recombinant *Pf*. conserved *Plasmodium* protein, *Pf*.RACK, *Pf*.TRP1 and *Pf*.WARP demonstrated gel mobility on SDS-PAGE at the predicted molecular weight of ~37 kDa, 38 kDa, 24 kDa and 32 kDa, respectively.

ELISA screening of culture supernatants of hybridomas against purified recombinant antigens resulted in identification of 16, 120, 19 and 21 hybridoma clones that produced antibody specific for *Pf*. conserved *Plasmodium* protein, *Pf*.RACK, *Pf*.TRP1 and *Pf*.WARP, respectively. The 10 highest ELISA reactive clones were then single cell cloned by limiting dilution. After a stringent selection process, the hybridoma clone that was most reactive for each recombinant protein was selected for antibody production by ascites (Envigo, Somerset, NJ) and subsequent IgG purification on a rProtein-A column. Monoclonal antibodies against *Pf*. conserved *Plasmodium* protein, *Pf*.RACK, *Pf*.TRP1 and *Pf*.WARP were named R3021, U8021, V4022 and Y3041, respectively.

### Relationship between mRNA transcription and protein translation of candidate detection markers during *P. falciparum* development in the mosquito

**Figure 2:**
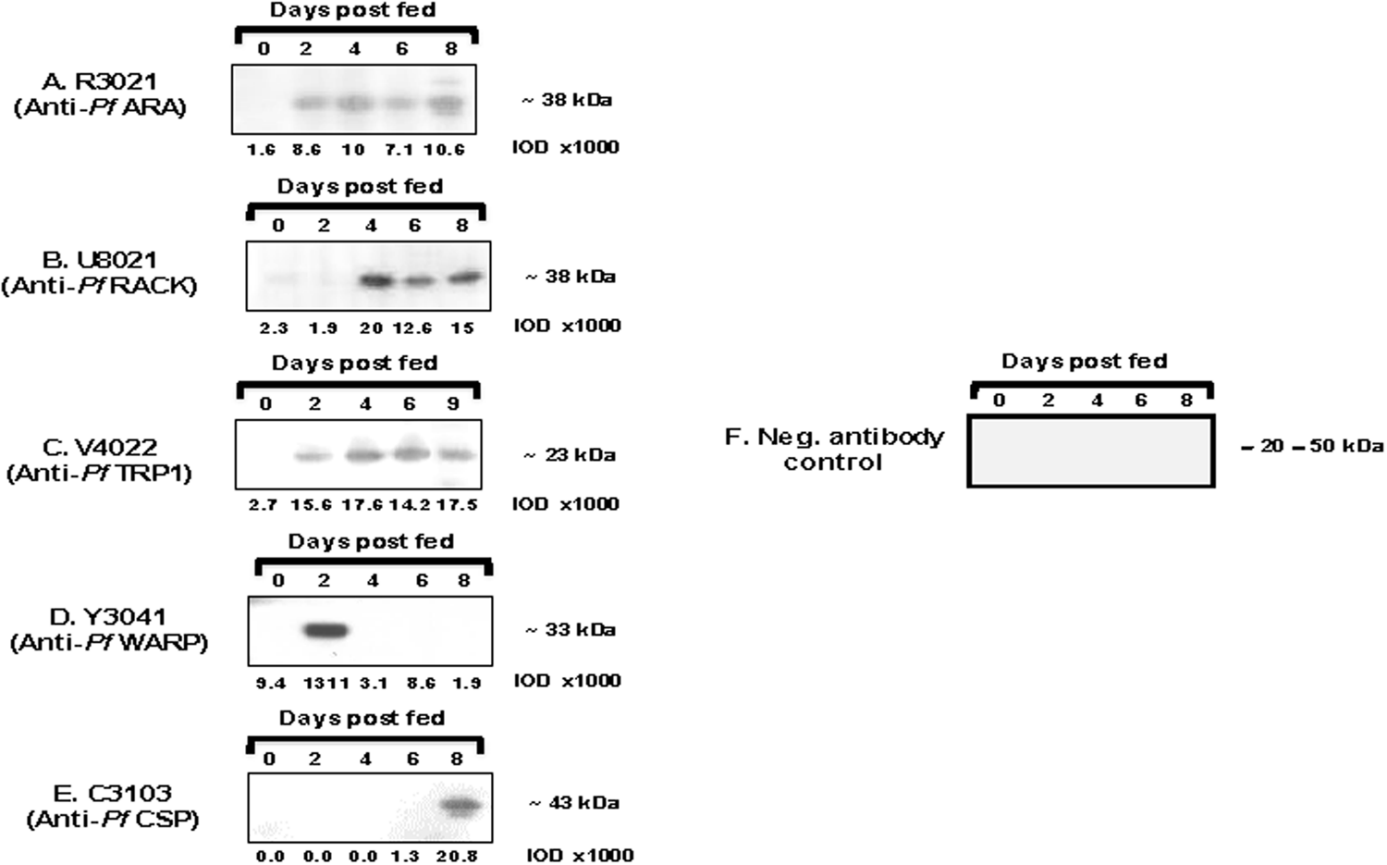
Measurement of protein expression of candidate detection markers by western blot on days 0, 2, 4, 6, 8 (9) post-blood meal in mosquitoes. Two oocysts were loaded per lane and *Pf*. conserved *Plasmodium* protein (**A**), *Pf*. receptor for activated C kinase homolog (RACK) (**B**), *Pf*. thioredoxin peroxidase 1 (TRP1) (**C**), *Pf*. von Willebrand factor A-domain-related protein (WARP) (**D**), *Pf*. circumsporozoite protein (**E**) and negative antibody control (**F**) were detected using monoclonal antibodies specific for the respective *Pf* recombinant proteins. Details of the secondary antibodies and Western blot reagents are provided in the Methods section. Expression levels were quantitated by measuring the intensities of protein bands in individual lanes using MetaMorph Software (version 6.1) and are represented as IOD units.

We next used monoclonal antibodies against *Pf*. conserved *Plasmodium* protein, *Pf*.RACK, *Pf*.TRP1 and *Pf*.WARP to determine whether these novel biomarkers can detect *P. falciparum* infection of mosquitoes by western blot analysis prior to day 8, the earliest time point that anti-*Pf*.CSP can detect an infection (Fig. 2a–e). Remarkably, monoclonal antibodies against *Pf*. conserved *Plasmodium* protein and *Pf*.TRP1 could detect *P. falciparum* infection of mosquitoes on days 2, 4, 6, and 8 (9) and anti-*Pf*.RACK could detect presence of *P. falciparum* parasites within the mosquito on days 4, 6, and 8 post-infection. For the *Pf*.WARP gene, there was a remarkable correlation between transcriptional and translational expression measured by qPCR and western blot, respectively; the kinetics of *Pf*.WARP gene expression in the mosquito was marked by robust transcription (204,000 copies) (Table 2) and translation (1311 × 10^3^ IOD) (Fig. 2d) on day 2 post-infection that was followed by a precipitous decline in expression by day 4 post-infection.

### Genetic diversity in the *P. falciparum* conserved *Plasmodium* protein, RACK, TRP1, and WARP antigens

We wanted to understand the distribution and natural dynamics of the polymorphisms in the four *P. falciparum* transmission stage antigens (*Pf*. conserved *Plasmodium* protein, *Pf*.RACK, *Pf*.TRP1, and *Pf*.WARP) in endemic populations where diversity is driven by naturally acquired immunity. To accomplish this, we compared the genetic variation of nucleotide sequences coding for the respective antigens for 202 *P. falciparum* field isolates available in Plasmodb.org. *Pf*. conserved *Plasmodium* protein exhibited 18 non-synonymous and 7 synonymous substitutions in all 202 isolates resulting in a non-synonymous/synonymous single nucleotide polymorphism (SNP) ratio of 2.57. *Pf*.RACK exhibited 2 non-synonymous and 3 synonymous substitutions resulting in a non-synonymous/synonymous SNP ratio of 0.67. *Pf*.TRP1 exhibited 0 non-synonymous and 3 synonymous substitutions resulting in a non-synonymous/synonymous SNP ratio of 0. Lastly, *Pf*.WARP exhibited 13 non-synonymous and 2 synonymous substitutions resulting in a non-synonymous/synonymous SNP ratio of 6.5. In summary, *Pf*.RACK and *Pf*.TRP1 had 2 and 0 non-synonymous substitutions, respectively, suggesting an absence of positive selection pressure on these antigens. However, *Pf*. conserved *Plasmodium* protein and *Pf*.WARP exhibited 18 and 13 non-synonymous substitutions in all 202 isolates, indicating the possibility of natural selection driven by immune pressure resulting in phenotypic changes.

### Effect of blood meal remnants on detection of *Pf*.RACK and *Pf*.TRP1 in the mosquito midgut

*Pf*.RACK and *Pf*.TRP1 are pan-developmentally expressed and highly conserved and therefore are promising candidates for an assay that could detect *P. falciparum* oocyst development in *A. stephensi* mosquitoes. We wanted to determine whether early expression of these proteins was oocyst specific or due to gametocyte or blood stage remnants in the blood meal. Therefore, we performed western blot at 6 hours, 24 hours (day 1), and 48 hours (day 2) on midgut samples collected from mosquitoes that were fed *Pf*. gametocytes versus 1) heat killed (56 °C for two hours) *Pf*. gametocytes (that cannot develop into oocysts and 2) blood stage parasites without gametocytes.

**Figure 3:**
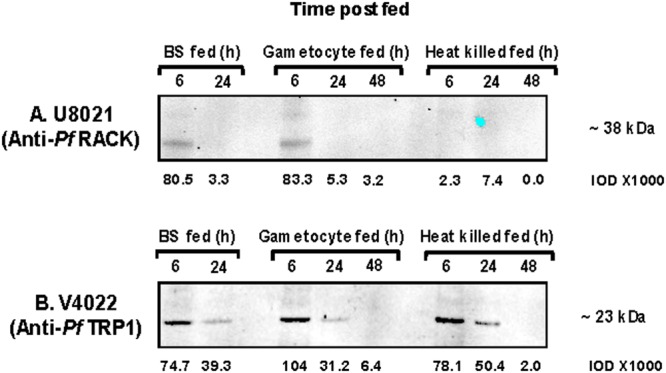
Effect of blood meal remnants on detection of *Pf*.RACK and *Pf*.TRP1 in the mosquito midgut. Protein expression of *Pf*. receptor for activated C kinase homolog (RACK) (**A**), and *Pf*. thioredoxin peroxidase 1 (TRP1) (**B**) was measured in midguts isolated from *Anopheles stephensi* mosquitoes at 6, 24, and 48 hours post blood meal with *P. falciparum* blood stage (BS), gametocyte, or heat killed gametocyte cultures. Samples equivalent to ten oocysts were loaded per lane and monoclonal antibodies specific for the respective *Pf* recombinant proteins were used as primary antibodies. Details pertaining to the secondary antibodies and Western blot reagents used can be found in the Methods section. Expression levels were quantitated by measuring the intensities of protein bands in individual lanes using MetaMorph Software (version 6.1) and are represented as IOD units.

For *Pf*.RACK, which was expressed in the mosquito midgut on days 4, 6, and 8 post-infection (Fig. 2), a band was present at 6 hours in midguts isolated from mosquitoes fed blood stage parasites and gametocytes. However, this band disappeared by 24 hours (Fig. 3). These results suggest that *Pf*.RACK expression at 6 hours is a consequence of gametocyte and blood stage remnants in the blood meal. However, gametocyte and blood stage specific *Pf*.RACK protein disappears by 24 hours post-blood meal indicating that *Pf*.RACK expression in the mosquito midgut on days 4, 6, and 8 post-infection is specific to the developing oocyst stage of the parasite.

For *Pf*.TRP1, protein was abundantly expressed (74.7 × 1000 IOD units) at 6 hours post-blood meal in midguts isolated from mosquitoes that were fed blood stage parasites. However, blood stage parasite specific *Pf*.TRP1 protein expression decreased 1.9 fold by 24 hours post-blood meal. Similarly, in midguts isolated from mosquitoes that were fed gametocytes, *Pf*.TRP1 was highly expressed (104 × 1000 IOD units) at 6 hours post-blood meal but decreased 3.3 fold from 6 to 24 hours and 4.9 fold from 24 to 48 hours. Similar results were observed in midguts isolated from mosquitoes that were fed heat killed gametocytes strongly suggesting that *Pf*.TRP1 protein expression at these timepoints is not induced by developmental progression from the gametocyte to the ookinete/oocyst but instead is a consequence of gametocyte and blood stage remnants in the blood meal. Importantly, the fact that *Pf*.TRP1 expression decreases significantly in all three types of samples over the 6 to 48 hour time period suggests that the probability of a “false positive” caused by blood meal remnants decreases as the parasite developmentally progresses through the mosquito.

## Discussion

The mosquito vector provides a relatively safe environment for the parasite to undergo sexual recombination, sporogenic differentiation and subsequent inoculation of infectious sporozoites into a vertebrate host. While the early developmental stages (gametocyte, gamete, zygote, and ookinete) have been shown to be targets of the human immune response through transmission-blocking antibodies, and hence attractive vaccine targets, the sporogenic cycle in the midgut oocyst is shielded from external intervention measures. In recent years, limited information has become available on the transcriptome/proteome of the gametocyte/gamete^11–13^, zygote, and ookinete^14,15^ stages of *P. falciparum*; the parasite genes associated with the developmental stages in the oocyst remain obscure. The oocyst stage is marked by rapid nuclear division and cellular differentiation leading to the production of sporozoites which eventually leave the midgut and travel to the salivary gland to attain their infectious form. In this study, we created a transcriptome map of the parasite during this stage of development by performing microarray studies on *Anopheles* mosquito midguts at days 2, 4, 6, and 8 post-*P. falciparum* infectious blood meal.

While our microarray results contain valuable information for future studies on oocyst developmental biology and possible transmission-blocking vaccine candidates, a major goal of this study was to identify novel parasite detection markers that can be used to monitor the developmental progression of oocysts in the *Anopheles* midgut. Currently, the standard membrane feeding assay (SMFA), a microscopy based assay that quantitates the number of oocysts that develop inside a mosquito midgut^16,17^, is used to determine *Plasmodium* burden on day 8 post-blood meal in mosquitoes. However, efforts are being made to replace microscopy-based detection with immunological assays that utilize the detection of CSP. A major limitation of both assays is that infectivity can only be detected after day 7 post-blood meal. Using a comprehensive approach that included identification of candidate detection markers by microarray, rigorous validation of the microarray dataset by qPCR, and subsequent evaluation of validated detection markers by western blot, we discovered a signature of genetic and protein markers for *P. falciparum* detection as early as day 2 post-infectious blood meal.

Our microarray studies revealed detectable expression of 212 genes as the *P. falciparum* oocysts undergo maturation (days 2, 4, 6 and 8 post-infectious blood meal) in the *A. stephensi* midgut. While expression of some of the genes was limited to a particular developmental stage, a majority of genes were expressed at more than one time point. Remarkably, expression of six genes was detectable as early as two days post-blood meal and three genes were found to be expressed at all time points measured. These results suggest the involvement of both developmentally regulated and pan-developmental genes in sporozoite formation.

In this study, we performed qPCR on 17% of the 212 statistically significant genes in our microarray dataset and noted that the correlation between fold change by microarray versus copy number by qPCR improved at later time points (Fig. 1). This could be a consequence of an increase in the parasite: vector RNA ratio as the parasite progresses developmentally through the mosquito. We next compared the expression of nine promising genes to *Pf*.CSP by qPCR on day 0 (uninfected), 6 hours, day 1, day 2, day 4, day 6, and day 8 post-*P. falciparum* infectious blood meal (Table 2). *Pf*.CSP has been shown to be required in successful malaria transmission from mosquito to mammalian host^18,19^. While previous studies report that day 7 is the earliest *Pf*.CSP can be detected by Western Blot in *P. falciparum* infected mosquitoes^10^, we find that *Pf*.CSP transcript can be detected as early as 6 hours post-infectious blood meal. However, three candidate genes (*Pf*.H2B, *Pf*.GAPDH, and *Pf*.TRP1) displayed transcriptional expression that was more abundant to *Pf*.CSP throughout the developmental stages of the oocyst with *Pf*.H2B exhibiting the highest level of expression. Additionally, although *Pf*.p23 and *Pf*.RACK transcript levels were lower than *Pf*.CSP on day 8 post-infection, RNA expression of both genes was significantly higher on days 1, 2, 4, and 6 post-infection compared to *Pf*.CSP.

Lastly, we compared the protein expression of *Pf*. conserved *Plasmodium* protein, *Pf*.RACK, *Pf*.TRP1, and *Pf*.WARP to *Pf*.CSP by western blot on days 2, 4, 6, and 8 (or 9) post-blood meal in mosquitoes. Robust expression of the *Pf*. conserved *Plasmodium* protein and *Pf*.TRP1 protein was observed throughout the developmental stages of the oocyst (days 2, 4, 6, and 8 (or 9)) whereas expression of *Pf*.WARP protein was detected on day 2 only and *Pf*.RACK was detectable on days 4, 6, and 8 only. However, nucleotide sequence analysis revealed a high level of conservation in *Pf*.RACK and *Pf*.TRP1 and a relatively high polymorphism in *Pf*. conserved *Plasmodium* protein and *Pf*.WARP. While *Pf*.RACK and *Pf*.TRP1 are cytosolic, *Pf*.WARP is expressed on the ookinete 12 hours post-fertilization^20^ and is a possible immune target subjected to immune pressure. The overall lack of or limited genetic diversity in these antigens makes them suitable biomarkers for detection of *P. falciparum* parasites undergoing development in the mosquito midgut. Based on these results, we propose that a detection assay utilizing the *Pf*.RACK and *Pf*.TRP1 proteins would offer superior sensitivity than a *Pf*.CSP-based assay of mosquito infection and would enable determination of infectivity throughout the parasite development in the mosquito vector. Furthermore, a multiplex assay that includes one of these pan-developmental expression proteins, an early detection marker such as *Pf*.WARP, and a late detection marker such as *Pf*.CSP would enable accurate discrimination between early versus late *Plasmodium* infection of mosquitoes in a single assay. An assay that captures all *Plasmodium* developmental stages would be particularly useful for assessing the rate of malaria transmission in mosquitoes and the impact of vector-based intervention measures (e.g., bed nets, drugs, vaccines, etc.) in endemic areas. In this study, we have identified a repertoire of molecules expressed during maturation of *P. falciparum* oocysts in the mosquito midgut. However, extensive validation will need to be performed to determine the applicability of these molecules as markers of early stage malaria infection in mosquitoes. Studies from different laboratories across Africa utilizing different species of *Anopheles* mosquitoes in the Standard Membrane Feed Assay and Direct Feed Assay would be needed to further validate these novel molecules as immunological biomarkers of malaria infection in mosquitoes.

Our studies also open the possibility to generate gene expression profiles in *Anopheles* species of different vectorial competence that produce varying oocyst counts with the same parasite strain to gain insight on developmentally regulated genes in successful sporozoite production and transmission.

## Methods

### *P. falciparum* Transmission into Mosquitoes and Oocyst Production

*P. falciparum* strain NF54 was maintained in continuous culture according to the method described by Trager and Jensen^21^. Briefly, *P. falciparum* was grown in O^+^ red blood cells (RBCs) at 4% hematocrit in RPMI 1640 medium supplemented with glutamine, HEPES, hypoxanthine, and 10% O^+^ human serum. Parasite cultures were maintained at 37 °C in an incubator. Use of human erythrocytes to support the growth of *P. falciparum* in cultures was approved by the internal review board of the Johns Hopkins Bloomberg School of Public Health. Gametocytemia and exflagellation events were assessed after 18 days of *P. falciparum* culture. The gametocyte culture was centrifuged and diluted in a mixture of 50% RBCs and human serum for membrane feeding. *Anopheles stephensi* mosquitoes were then fed on membrane feeders for 30 min with blood containing 0.3% mature *P. falciparum* gametocytes. The fed mosquito cage was then sorted and unfed mosquitoes were removed. At 6 hr, 1, 2, 4, 6 and 8 days post infectious blood feeding, the fed mosquitoes were dissected and following mercurochrome staining the oocysts counts were determined by microscopy on day 8 post-infection. For a representative experiment (n = 36 mosquitoes), the prevalence of *P. falciparum* infection of *A. stephensi* mosquitoeswas 91.7% and the median number of oocysts per midgut was 31.5 (Supplemental Figure S1).

### Preparation of RNA

High quality RNA was prepared from 20 pooled uninfected or infected mosquito midguts (approximately 64 oocysts per midgut) using Trizol^®^ Reagent (Invitrogen, Carlsbad, CA). Briefly, mosquito midguts were homogenized in 1 mL of Trizol^®^ Reagent for immediate stabilization of RNA, snap frozen on dry ice, and stored at −80 °C. For RNA extraction, samples were briefly thawed and RNA was precipitated with isopropanol, washed with 70% ethanol, and then resuspended in nuclease-free water. Quality and quantity of RNA was assessed using the Nanodrop 2000 spectrophotometer. For microarray experiments, RNA was prepared from uninfected and infected mosquito midguts on days 2, 4, 6, and 8 post-infection. For quantitative real time PCR (qPCR) experiments, RNA was prepared from uninfected and infected mosquito midguts at 6 hours and days 1, 2, 4, 6, and 8 post-infection.

### Microarray Studies

Microarray studies were performed in quadruplicate comparing RNA isolated from uninfected (day 0) versus *P. falciparum* infected (days 2, 4, 6, and 8) mosquito midguts to identify candidate genes that could be used to detect malaria transmission by mosquitoes. Amplification of RNA was performed to generate a sufficient quantity for microarray as previously described^22^. Briefly, the Low Input QuickAmp Labeling Kit (Agilent Technologies, Santa Clara, CA) was used for RNA amplification and incorporation of label. Briefly, cDNA was synthesized from 300 ng of RNA template at 40 °C for 2 hours in a reaction mixture containing AffinityScript-Reverse Transcriptase, Oligo dT-Promoter Primer, DTT, and dNTPs. Next, amplified labeled cRNA was generated from cDNA at 40 °C for 2 hours in a reaction mixture containing T7 RNA polymerase, DTT, NTPs, and either cy3 (uninfected mosquito midgut) or cy5 (*P. falciparum* infected mosquito midgut) label. After amplification and incorporation of label, labeled cRNA transcripts were washed twice to remove unincorporated label and resuspended in RNase-free water using the RNeasy^®^ Mini Kit (Qiagen, Valencia, CA). Amplified labeled cRNA products were then quantified using the Nanodrop 2000 and importantly, equivalent amounts of cy3 (uninfected mosquito midguts) and cy5 (*P. falciparum* infected mosquito midguts) labeled cRNA were combined in a single tube. This sample containing equal amounts of cy3 and cy5 labeled cRNA was concentrated using a Vivaspin 500 column (Sartorius Stedim Biotech, Goettingen, Germany) to an appropriate loading volume (20 μl) for hybridization to a custom-designed Agilent SurePrint oligonucleotide array (Agilent Technologies) with 44,000 features (including 5,254 distinct 60-mer probes for Pf 3D7 transcripts, each spotted 8 times). After hybridization, the microarray chip was scanned at 5 micron resolution and image-analyzed using Feature Extraction software version 9 and data was filtered using NIAID microarray database tools (http://mAdb.niaid.nih.gov) as previously described^23–26^.

### Quantitative real-time PCR

Quantitative real time PCR was performed as previously described^27^ to validate results obtained by microarray. RNA was first treated with 4 U of Turbo DNA-free (Ambion, Austin, TX) for 30 minutes at 37 °C, and cDNA was then synthesized from 5 μg of DNase-treated RNA in a reaction containing superscript II RNaseH^−^ reverse transcriptase, random hexamers, deoxynucleotide triphosphates (dNTPs), dithiothreitol, and magnesium chloride (Invitrogen, Carlsbad, CA) using the following cycle profile: 25 °C for 10 min, 42 °C for 50 min, 72 °C for 7 min, and then cooled to 4 °C. After cDNA synthesis, the product was treated with RNAse H at 37 °C for 30 min to degrade residual RNA template. Quantitative real time PCR was then performed in a 20 μl reaction volume containing 2 μl cDNA (diluted 1:5), 12.5 μl iQ SYBR Green Supermix, and 10 μmol primers. Amplification and detection of specific product were performed using the CFX96 Touch Real-Time PCR detection system (Biorad Laboratories, Hercules, CA) with the following cycle profile: 1 cycle at 94 °C for 30 sec and 35 cycles with 1 cycle consisting of 5 sec of denaturation at 94 °C and 5 sec of annealing and extension at 59 °C. The relative concentrations of RNA were determined using a standard curve derived from the PCR products of 10-fold serial dilutions of plasmid containing a *P. falciparum* β-actin gene fragment. Real time PCR was performed on two sets of mosquito midgut preparations in triplicate. Primers used for validation of microarray biomarkers can be found in Supplemental Table S2.

### Protein expression and purification

Expression of *Pf*. conserved *Plasmodium* protein (PF3D7_0931300), *Pf*.RACK (Pf3D7_0826700), *Pf*.TRP1 (PF3D7_1438900), and *Pf*.WARP (PF3D7_0801300) recombinant proteins were accomplished by amplifying the full length gene using primers listed in Supplemental Table S3. The putative signal and transmembrane sequences were identified using SignalP 4.1 Server and TMHMM Server v. 2.0, respectively, excised, and the in domain region was then selected for recombinant expression. The PCR-amplified product was next cloned into the *Not*I and *Asc*I (NEB, Ipswich, MA) restriction sites in a pET11a vector (MERCK, Germany) which was modified to include an NH2-terminal hexa-histidine tag to facilitate purification. The protein expression was carried out in *E. coli* BL-21 (λDE3) cells with IPTG induction. Induced *E. coli* cells were lysed with BugBuster Protein Extraction Reagent (EMD Millipore, Billerica, MA) and the soluble proteins were purified from the supernatant on a HisTrap column (GE Healthcare life sciences, Pittsburgh, PA). The insoluble proteins were purified by lysing the cell pellet with a combination of lysozyme and sonication, followed by 4–6 washes with buffer (50 mM Tris pH 8.0, 20 mM EDTA) to obtain pure inclusion bodies (IBs). The insoluble protein in the IBs was denatured in solubilization buffer (0.1 M Tris pH 8.0, 2 mM EDTA, 6 M Guanidine HCl) before refolding under controlled redox condition in renaturation buffer (0.1 M Tris pH 8.0, 2 mM EDTA, 0.5 M L-Arginine HCl, 0.9 mM oxidized Glutathione). The refolded protein was then dialyzed against a gradient of urea and finally brought into 20 mM Tris pH 8.0 buffer and purified on a HisTrap column. The purified recombinant proteins were quantified using Bradford’s reagent (Sigma-Aldrich, MO) and the quality as well as purity was checked on SDS-PAGE followed by Coomassie blue staining.

### Immunization and monoclonal antibody production

Female Balb/c mice (5–6 weeks old) were purchased from Jackson Laboratories (Bar Harbor, ME) and were maintained at the Center for Biologics Evaluation and Research (CBER) animal care facility and treated in accordance and under the guidelines of the CBER Animal Care and Use Committee. All studies in mice were performed under animal study protocol number 2014-06 which was reviewed and approved by the CBER Animal Care and Use Committee at FDA. Mice (5 per group) were immunized three times with 50 μg of 3D7 *Pf*. conserved *Plasmodium* protein, *Pf*.RACK, *Pf*.TRP1 or *Pf*.WARP purified recombinant protein per animal intraperitoneally in Freund’s adjuvant (Complete Freund’s adjuvant for the primary dose followed by two booster doses in Incomplete Freund’s adjuvant) at three weeks intervals. Serum samples were collected 10 days after the third immunization and titrated against the respective immunized recombinant antigens. After three weeks post third immunization, two of the highest titered mice from each group were further boosted intravenously with 20 μg of recombinant antigen in phosphate-buffered saline (PBS). Three to five days post last immunization, mice were euthanized and spleens were harvested aseptically inside the culture hood. The fusion of the antibody secreting splenocytes with Sp2/0 Ag14 myeloma cells was essentially carried out following the procedure developed by Köhler and Milstein^28^. After fusion, the hybridoma cells were grown in HAT (hypoxanthine-aminopterin-thymidine) selection medium and incubated at 37 °C with 5% CO_2_. About 7–8 days following fusion, supernatant from the wells were screened for the presence of antibodies in ELISA against the recombinant antigens. The hybridomas from the antibody positive wells were cloned by the limiting cell dilution method to obtain single cell clones secreting single specificity antibody against the corresponding antigen. Two of the highest reactive monoclonal antibody secreting hybridomas were selected against each antigen for ascites production to obtain a large amount of mAb. The mAb in the ascites were purified through column chromatography using rProtein-A beads (HiTrap rProtein-A column; GE Healthcare, Princeton, NJ).

### Western blot analysis

To prepare protein sample for western blot analysis, uninfected and *Pf* infected *A. stephensi* midguts with estimated oocyst intensities were first pulse-homogenized in phosphate buffered saline containing 1% sarkosyl and then centrifuged at 13,000 rpm for 2 minutes. Supernatant was then diluted to one oocyst per μl in SDS lysis buffer and boiled for 5 minutes prior to gel loading. *P. falciparum* lysates equivalent to two oocysts were loaded per well and *Pf*. conserved *Plasmodium* protein, *Pf*.RACK, *Pf*.TRP1, and *Pf*.WARP proteins were detected using monoclonal antibodies specific for the respective *Pf*. recombinant proteins and a commercially obtained chemiluminescence-linked Western blotting kit (Western Light Tropix, Bedford, MA). Protein bands were observed after incubation with enhanced chemiluminescence detection reagents, and the integrated optical densities (IOD) for each lane was measured using ImageJ software.

### Genetic Diversity

The presence of single nucleotide polymorphisms (SNPs) in *Pf*. conserved *Plasmodium* protein, *Pf*.RACK, *Pf*.TRP1, and *Pf*.WARP was studied by aligning the nucleotide sequences coding for the respective antigens with the 202 *P. falciparum* isolates available on Plasmodb.org. The presence of genetic variability within a gene resulting in phenotype change is measured by the number of non-synonymous substitutions.

### Statistical analysis

Correlation was calculated using the Spearman’s ρ nonparametric test. Error bars indicate standard deviation for qPCR. The Student’s *t* test was used to calculate significant fold change for microarray studies.

### Data availability

All data generated or analysed during this study are included in this published article (and its Supplementary Information files).

## Electronic supplementary material

Supplemental Information

Supplemental Table S1
