# Supplementary material for: Transcriptome analysis based detection of Plasmodium falciparum development in Anopheles stephensi mosquitoes
Source: Sci Rep. 2018 Aug 1;8:11568. doi: 10.1038/s41598-018-29969-4 (PMC6070505; doi:10.1038/s41598-018-29969-4)
Supplement: Supplementary file 1 — Supplemental Information [file 41598_2018_29969_MOESM1_ESM.pdf]

Supplemental information

Transcriptome analysis based detection of *Plasmodium falciparum* development in *Anopheles stephensi* mosquitoes

Miranda S. Oakley, Nitin Verma, Timothy G. Myers, Hong Zheng, Emily Locke, Merribeth J. Morin, Abhai K. Tripathi, Godfree Mlambo and Sanjai Kumar

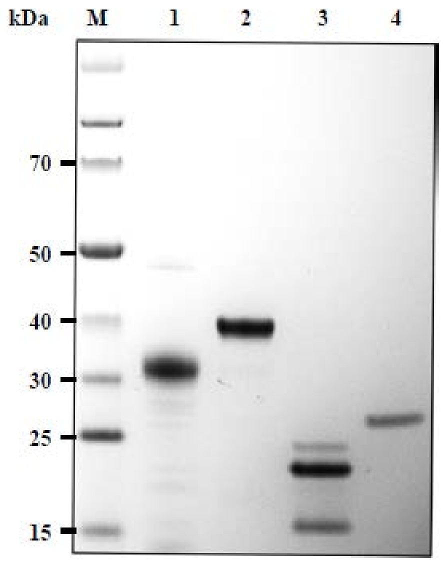

Figure S1. SDS-PAGE analysis of His-tag purified, recombinant *Pf*.WARP, *Pf*.RACK, *Pf*.TRP1 and *Pf*. conserved *Plasmodium* protein. Proteins were separated on 4-12% SDS-PAGE gradient under reducing conditions and stained with Simply Blue Safestain. Lane M, Molecular weight marker; Lane 1, *Pf*.WARP; Lane 2, *Pf*.RACK; Lane 3, *Pf*.TRP1; Lane 4, *Pf*. conserved *Plasmodium* protein.

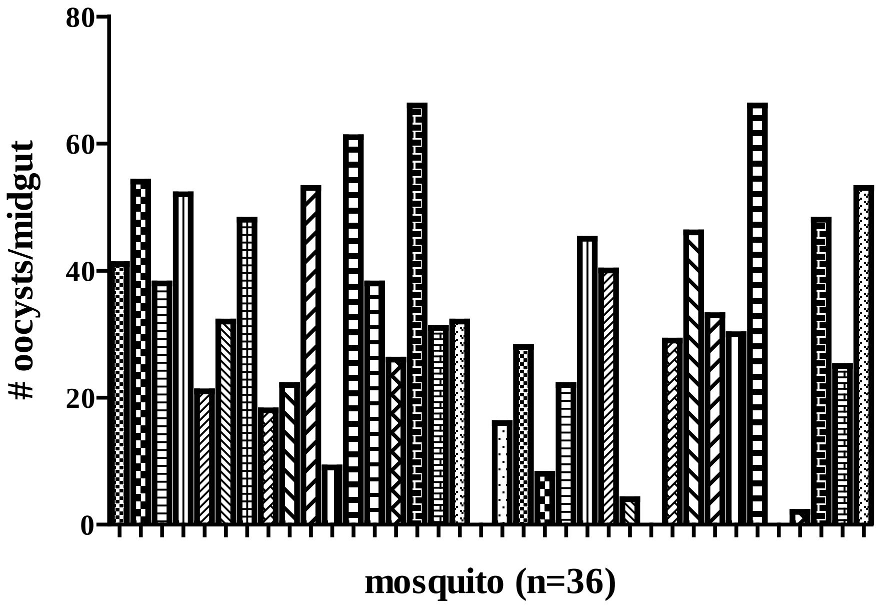

Figure S2. Prevalence and oocyst burden of a representative *P. falciparum* infection of *A. stephensi* mosquitoes. On day 8 post-infection, fed mosquitoes (n=36) fed mosquitoes were dissected and stained with mercurochrome and oocysts counts were then determined by microscopy.

Table S2. Primers used for quantitation of RNA transcript by qPCR.

Gene	PlasmoDB ID	Forward Primer	Reverse Primer
**Conserved**	PF3D7_0931300	ATGAAGGGCAAAGTTGTTGA	TTATTCGGTACCCGTCAAAC
**CSP**	PF3D7_0304600	CACAATATGCCAAATGACCC	AGGTTTATTAGCAGAGCCAG
**DHFS-FPGS**	PF3D7_1324800	AGCTACCGAGCAAATACAAC	TCAAGTTTCTCGGCTTTGTT
**ALBA1**	PF3D7_0814200	ACCAGATGTGGAAGTACAGT	CCTCTTCTGAATGGCCTAGA
**GAPDH**	PF3D7_1462800	AACAGGTGTAGCTTTCAGAG	GCTAAACCAGCTTTCATGTC
**H2B**	PF3D7_1105100	CACCCAGATACTGGTATTTCA	GTTACAGCTTTGGTTCCTTC
**P23**	PF3D7_1453700	GCTGGATTAGGTGGTATGGA	ACTGCTGCATCAGGAATTTT
**RACK**	PF3D7_0826700	GTATCAACACCAACAGACCC	TGGTCCCAAGAACCTGATAA
**TRP1**	PF3D7_1438900	AGGCTGTAGTGTGGATAGTA	GTTGAACAACACCTTGCTTG
**WARP**	PF3D7_0801300	GGCTCATATGTCAGTTGTCC	TTAGGAGCATCCTTTCGAGT

Table S3. Primers sets used for the amplification of *Pf.*WARP, *Pf.*RACK, *Pf.*TRP1 and *Pf.* conserved *Plasmodium* protein antigenic domains used for recombinant expression in *E. coli*.

***P. falciparum* antigen**	**Antigenic domain**	**Primers set**
*Pf.*WARP	N_{26}-S_{290}	5′primer: 5′- ATACTATCGCGGCCGCTAACGTAGTGTCTCATAACTCTATG-3′<br>3′primer: 5′- ATAGTATAGGCGCGCCTGATTTATTCTTATCACATATTTTTTGA-3′
*Pf.*RACK	M_2-V_{323}	5′primer: 5′- ATACTATCGCGGCCGCTATGGATAATATAAAAGAAGCTG-3′<br>3′primer: 5′- ATAGTATAGGCGCGCCAACTGAGTGTTTTTTAACTTCATATAC-3′
*Pf.*TRP1	A_2-L_{195}	5′primer: 5′ ATACTATCGCGGCCGCTGCATCATATGTAGGAAGAGAAGC-3′<br>3′primer: 5′- ATAGTATAGGCGCGCCCAACTTTGATAAATATTCACTAAC-3′
Pf. conserved *Plasmodium* protein	M_1-E_{305}	5′primer: 5′ ATACTATCGCGGCCGCTATGCTTTCCATAGCAAGTACCTTC-3′<br>3′primer: 5′- ATAGTATAGGCGCGCCTTCATTTTTGTTTATTTCCTCATC-3′
